# Supplementary material for: Generative modeling of multi-mapping reads with mHi-C advances analysis of Hi-C studies
Source: eLife. 2019 Jan 31;8:e38070. doi: 10.7554/eLife.38070 (PMC6450682; doi:10.7554/eLife.38070)
Supplement: Supplementary file 1. [file elife-38070-supp1.pdf]

Table 1. Hi-C and mHi-C terminology.

Terminology	Description	Diagram	Standard Pipeline	mHi-C
1a. Read ends alignment	Each end of a paired-end read is aligned independently.	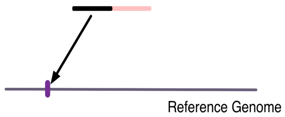	Uni-reads	Uni&multi-reads
1b. Chimeric read rescue	Read end spans ligation site. They are rescued by trimming and re-alignment.	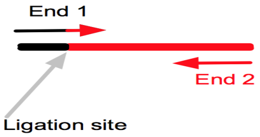	Uni-chimeric ends	Uni&multi-chimeric ends
2a. Read end pairing	Aligned read ends form an alignment position pair.	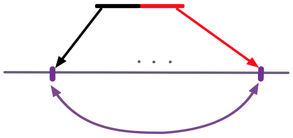	Uni-read pairs	Uni&multi-read pairs
2b. Multi-reads reduced to uni-reads due to validation checking	Only one of the alignment position pairs passes the validation checking.	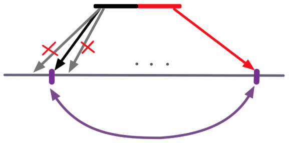	Valid uni-reads	Valid uni- and multi-reduced uni-reads
3a. Bin pair	Bin the genome by a fixed size window. Read end alignments fall within bins and form bin pairs indicating contact from one bin to another.	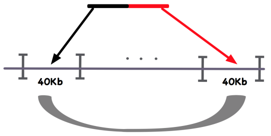	Uni-reads binning	Uni&multi-reads binning
3b. Multi-reads reduced to uni-reads due to binning	Each read end has multiple alignment positions falling within the same bin, thus supporting the same bin pair.	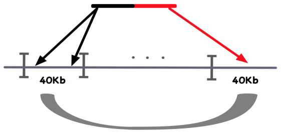	Uni-reads bin pairs	Uni- and multi-reduced uni-bin pairs
4. Contact count	Each read pair alignment represents one contact. Contact count for bin pair j and k is the total number of read pairs with ends in bin_j and bin_k.	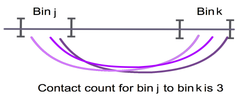	Uni-mapping contacts	Uni&multi-mapping contacts
5. Contact matrix	The entry at row j and column k indicates the contact count of bin_j interacting with bin_k.	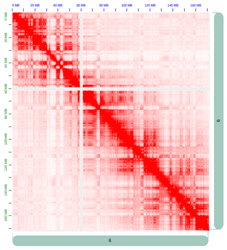	Uni-setting contact matrix	Uni&Multi-setting contact matrix
