# Supplementary material for: Generative modeling of multi-mapping reads with mHi-C advances analysis of Hi-C studies
Source: eLife. 2019 Jan 31;8:e38070. doi: 10.7554/eLife.38070 (PMC6450682; doi:10.7554/eLife.38070)
Supplement: Transparent reporting form [file elife-38070-transrepform.docx]

***eLife’s* transparent reporting form**

We encourage authors to provide detailed information *within their submission* to facilitate the interpretation and replication of experiments. Authors can upload supporting documentation to indicate the use of appropriate reporting guidelines for health-related research (see [EQUATOR Network](http://www.equator-network.org/%20)), life science research (see the [BioSharing Information Resource](https://biosharing.org/)), or the [ARRIVE guidelines](http://www.plosbiology.org/article/info:doi/10.1371/journal.pbio.1000412) for reporting work involving animal research. Where applicable, authors should refer to any relevant reporting standards documents in this form.

If you have any questions, please consult our Journal Policies and/or contact us: [editorial@elifesciences.org](mailto:editorial@elifesciences.org).

**Sample-size estimation**

- You should state whether an appropriate sample size was computed when the study was being designed
- You should state the statistical method of sample size computation and any required assumptions
- If no explicit power analysis was used, you should describe how you decided what sample (replicate) size (number) to use

Please outline where this information can be found within the submission (e.g., sections or figure legends), or explain why this information doesn’t apply to your submission:

This information does not apply to our manuscript since the method we developed utilizes existing datasets and presents an alternative analysis where multi-mapping reads are taken into account.

**Replicates**

- You should report how often each experiment was performed
- You should include a definition of biological versus technical replication
- The data obtained should be provided and sufficient information should be provided to indicate the number of independent biological and/or technical replicates
- If you encountered any outliers, you should describe how these were handled
- Criteria for exclusion/inclusion of data should be clearly stated
- High-throughput sequence data should be uploaded before submission, with a private link for reviewers provided (these are available from both GEO and ArrayExpress)

Please outline where this information can be found within the submission (e.g., sections or figure legends), or explain why this information doesn’t apply to your submission:

We utilized eight major existing Hi-C datasets of different cell types from six studies and a number of datasets from the ENCODE portal and GEO as supporting data. The details of the datasets from GEO are summarized in Figure1-sourceData. The accession numbers for the ENCODE and GEO datasets are in Section “ChIP-seq analysis” under “Materials and Methods”.

We only consider Hi-C interactions that are 25kb away and within 10Mb for human/mouse cell lines and 20kb away without any upper bound for *Plasmodium falciparum*. The majority of contacts in short range distance are random interactions which are not our analysis target and beyond 10Mb, there are fewer real interaction signals and mainly noise. Such Hi-C interaction exclusion rule has been widely adapted in standard Hi-C analysis pipelines (Ay et al 2014a, Servant et al., 2015).

**Statistical reporting**

- Statistical analysis methods should be described and justified
- Raw data should be presented in figures whenever informative to do so (typically when N per group is less than 10)
- For each experiment, you should identify the statistical tests used, exact values of N, definitions of center, methods of multiple test correction, and dispersion and precision measures (e.g., mean, median, SD, SEM, confidence intervals; and, for the major substantive results, a measure of effect size (e.g., Pearson's r, Cohen's d)
- Report exact p-values wherever possible alongside the summary statistics and 95% confidence intervals. These should be reported for all key questions and not only when the p-value is less than 0.05.

Please outline where this information can be found within the submission (e.g., sections or figure legends), or explain why this information doesn’t apply to your submission:

Statistical methods and analysis approaches are reported as parts of the Materials and Methods section. Relevant figures are referenced when appropriate results from statistical tests are reported within the text and the raw data presented in the figures are summarized into table format in Appendix 1 or appended as source data.

(For large datasets, or papers with a very large number of statistical tests, you may upload a single table file with tests, Ns, etc., with reference to sections in the manuscript.)

**Group allocation**

- Indicate how samples were allocated into experimental groups (in the case of clinical studies, please specify allocation to treatment method); if randomization was used, please also state if restricted randomization was applied
- Indicate if masking was used during group allocation, data collection and/or data analysis

Please outline where this information can be found within the submission (e.g., sections or figure legends), or explain why this information doesn’t apply to your submission:

This does not apply to this manuscript since the manuscript did not generate new data but utilized existing public data.

**Additional data files (“source data”)**

- We encourage you to upload relevant additional data files, such as numerical data that are represented as a graph in a figure, or as a summary table
- Where provided, these should be in the most useful format, and they can be uploaded as “Source data” files linked to a main figure or table
- Include model definition files including the full list of parameters used
- Include code used for data analysis (e.g., R, MatLab)
- Avoid stating that data files are “available upon request”

Please indicate the figures or tables for which source data files have been provided:

We uploaded source data for all the main figures. In addition, the software is available on GitHub with both documentation and example that runs through the whole pipeline. Other supplementary scripts utilized to compare models are also appended.

In addition, Figure 3 - Source data is available at

[http://datadryad.org/review?doi=doi:10.5061/dryad.v7k3140](http://datadryad.org/review?doi=doi:10.5061/dryad.v7k3140)
